# Solar spectra datasets at optimum and vertical installation angles in central Europe (Berlin) during 2020, 2021 and 2022

**DOI:** 10.1016/j.dib.2023.109273

**Published:** 2023-05-30

**Authors:** Guillermo A. Farias-Basulto, Maximilian Riedel, Mark Khenkin, Rutger Schlatmann, Reiner Klenk, Carolin Ulbrich

**Affiliations:** a PVcomB/Helmholtz-Zentrum Berlin für Materialien und Energie GmbH, Schwarzschildstr.3, Berlin D-12489, Germany; b HTW Berlin - University of Applied Sciences, Wilhelminenhofstr. 75a, Berlin D-12459, Germany

**Keywords:** Photovoltaic, Energy, Facade, Spectrum, Outdoor, Irradiance

## Abstract

This article provides datasets containing three years worth of solar spectra for the optimum installation angle of 35° and the building-integrated-photovoltaics relevant vertical angle of 90°. These datasets were obtained by measuring the spectrally resolved solar spectra using a five minute interval, where two sets of spectrometers, which measure different ranges of the solar spectrum, were employed. In addition, a merged dataset of these two spectral measurements, related to every specific five minute interval measurement, is provided. An analysis and interpretation of the data using only year the 2020 is provided in “Measurement and analysis of annual solar spectra at different installation angles in central Europe” [1].

**Specifications Table**

**Table:** 

Subject	Radiation
Specific subject area	Spectrally resolved solar radiation on a tilted surface
Type of data	Table
How the data were acquired	Two sets of spectrometer sensors from “eko-instruments” (MS-711 and MS-712) were installed in PVcomB/Helmholtz Zentrum Berlin (52°25′52.5″N 13°31′25.7″E) at two different angles of 35° and 90°. The spectrometer sensors provide a spectrally resolved measurement of the incident solar spectrum, each providing different ranges. The MS-711 has a measurement range of 300–1100 nm and the MS-712 has a measurement range of 900–1700 nm. The sensors have an optical resolution (FWHM) better than 7 nm with a wavelength accuracy of ±0.2 nm. The MS-711 has a temperature dependency below ±2% within an operating temperature of −10 to 50 °C, whereas the MS-712 has a temperature dependency of ±5% within a working temperature of −10 °C and 40 °C. The temperature is controlled at 25 °C (±2 °C) within an ambient temperature range from −10 °C to about 40 °C and between 25 °C and 32 °C above an ambient temperature of 40 °C and below 50 °C. Each measurement is controlled automatically, with an exposure time from 10 ms to 5000 milliseconds and a field of view of 180°. The dome material of the MS-711 is synthetic quartz, whereas BK7 glass is used for the dome of the MS-712 sensor. Calibration certificates issued by the manufacturer indicate combined uncertainties (including cosine and temperature dependency) for MS-711 of 17.4% (300–350 nm), 5.1% (350–450 nm), 4.2(450–1050 nm) and 5.3% (1050–1100 nm). For the MS-712, uncertainties of 4.5% (900–950 nm), 4.84% (950–1600 nm) and 23.67% (1600–1700 nm) were indicated. The spectrometer measurements per angle are automatically merged through the software provided by the manufacturer (i.e. WSDac, WSDisp) [2], which provides a new spectrum per measurement with a wavelength range of 300–1700 nm.
Data format	Raw
Description of data collection	No preconditioning of any kind was employed. This means that the data was collected completely under outdoor conditions and is presented as measured. The periods in which the light is below the sensitivity range of the spectrometers is also not included.
Data source location	• PVcomB/Helmholtz Zentrum Berlin • Berlin • Germany • 52°25′52.5″N 13°31′25.7″E
Data accessibility	Repository name: HZB Data Service Data identification number: https://doi.org/10.5442/ND000010. Direct URL to data: https://data.helmholtz-berlin.de/pub/ND000010 No access control required.

## Value of the Data

• The solar spectral data for two different angles have not been reported up to now.
• After integration of each solar spectrum, the data can be used as an input for calculations regarding yearly PV energy yield [3]. For this, PV characteristics such as spectra response and photovoltaic efficiency are needed.
• The data can be used to compare spectrally resolved irradiances at two different angles.
• Research institutes, universities, companies and private users in the field of photovoltaics can benefit from these data.
• The data can be evaluated for specific use cases by applying photon energy-material relations (e.g. Solar cell spectral response) [4].

## 1 Objective

**Fig. 1:**
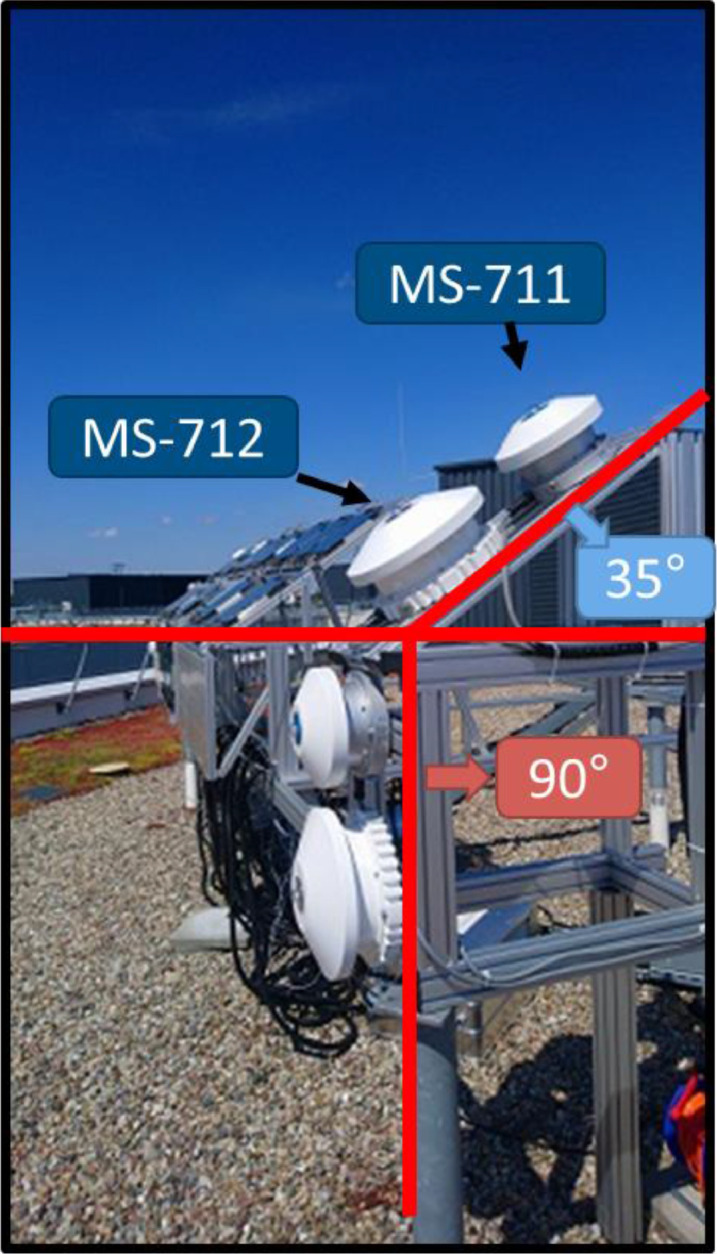
Picture of the installation of two sets of spectrometers MS-711 and MS-712 at two different tilt angles: 35° and 90°.

The objective of the data is to provide insights regarding solar spectral differences between the optimum angle of 35°, at which most installations at the presented location are installed, and the building integrated photovoltaics (BIPV), as these installations are often found in a vertical orientation. Fig. 1 shows a picture of the outdoor facilities, where the two spectrometers at an optimum tilt angle of 35° degrees and two at 90° (all facing south) can be seen. For the 35° installation angle, the two spectrometers are labeled.

## 2 Data Description

**Fig. 2:**
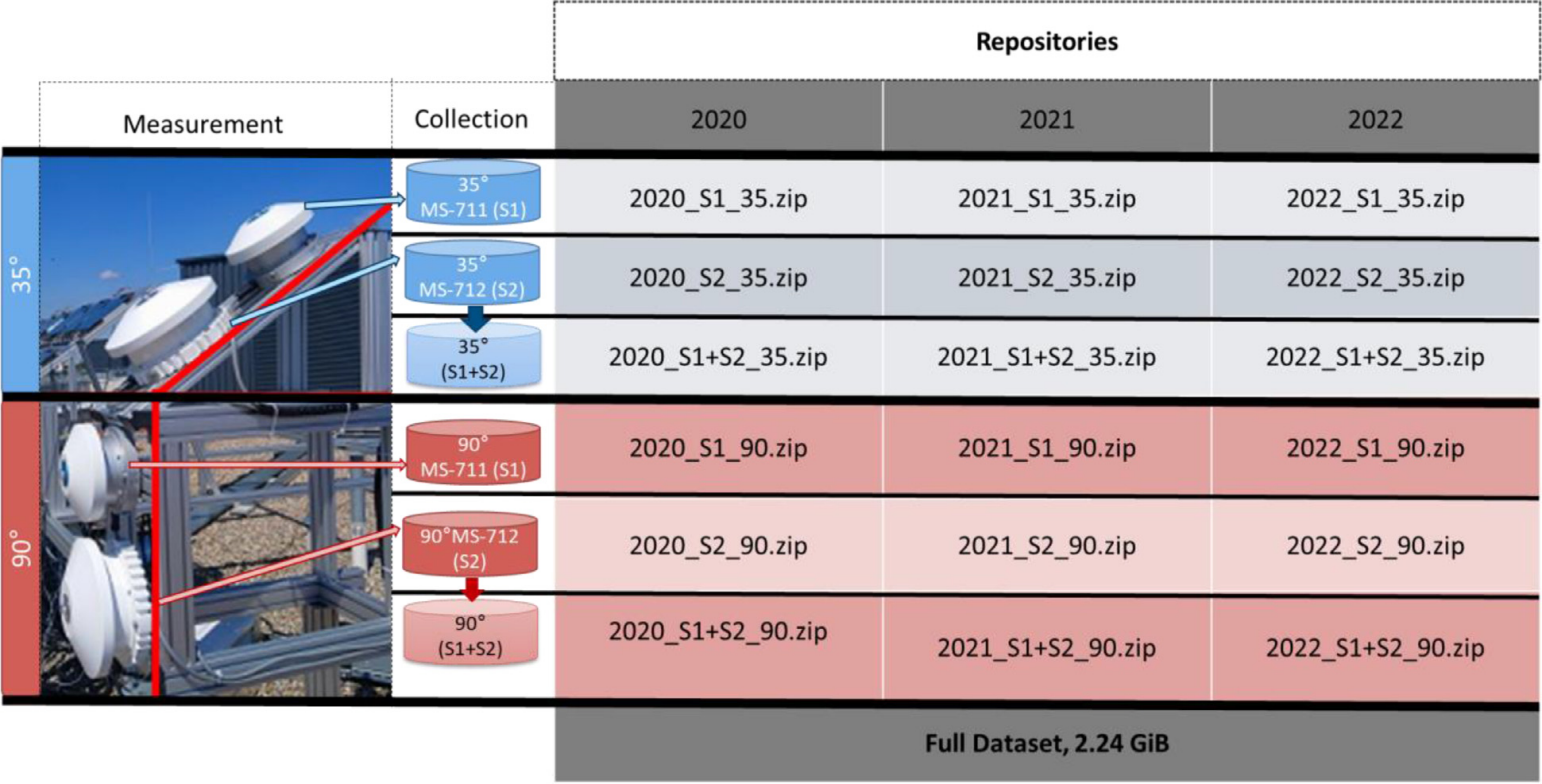
Data acquisition/collection and storage per spectrometer.

The datasets provide solar spectra for two installation angles: for an optimum installation angle of 35° and for a vertical angle of 90, relevant for building-integrated-photovoltaics. The datasets are provided as compressed raw data as measured by the MS-711 (300–1100 nm) spectrometers (S1) and by the MS-712 (900–1700 nm) spectrometers (S2). The merged data measured by MS-711 and 712 (S1+S2) is also provided. Fig. 2. Shows a flow chart of the data measurement and collection. Due to the size of the three-year datasets, we divided the whole datasets in three smaller compressed packages (.zip) per angle and per year, where each .zip file contains at least two csv files per year as described in Table 1.

**Table 1:** Raw data description per file provided.

Year	Angle	File name	Description
2020	35	2020_S1_35.zip	Data measured by MS-711 (300–1100 nm) during 2020 at 35° divided in three files: Jan to Mar; Apr to Jul; Aug to Dec.
		2020_S2_35.zip	Data measured by MS-712 (900–1700 nm) during 2020 at 35° divided in three files: Jan to Mar; Apr to Jul; and Aug to Dec.
		2020_S1+S2_35.zip	Merged data measured by MS-711 and 712 (300–1700 nm) during 2020 at 35° divided in three files: Jan to Mar; Apr to Jul; and Aug to Dec.

2020	90	2020_S1_90.zip	Data measured by MS-711 (300–1100 nm) during 2020 at 90° divided in three files: Jan to Mar; Apr to Jul; and Aug to Dec.
		2020_S2_90.zip	Data measured by MS-712 (900–1700 nm) during 2020 at 90° divided in three files: Jan to Mar; Apr to Jul; and Aug to Dec.
		2020_S1+S2_90.zip	Merged data measured by MS-711 and 712 (300–1700 nm) during 2020 at 90° divided in three files: Jan to Mar; Apr to Jul; and Aug to Dec.

2021	35	2021_S1_35.zip	Data measured by MS-711 (300–1100 nm) during 2021 at 35° divided in three files: Jan to Jun; and Jul to Dec.
		2021_S2_35.zip	Data measured by MS-712 (900–1700 nm) during 2021 at 35° divided in two files: Jan to Jun; and Jul to Dec.
		2021_S1+S2_35.zip	Merged data measured by MS-711 and 712 (300–1700 nm) during 2021 at 35° divided in two files: Jan to Jun; Jul to Sep; and Oct to Dec.

2021	90	2021_S1_90.zip	Data measured by MS-711 (300–1100 nm) during 2021 at 90° divided in two files: Jan to Jun; and Jul to Dec.
		2021_S2_90.zip	Data measured by MS-712 (900–1700 nm) during 2021 at 90° divided in two files: Jan to Jun; and Jul to Dec.
		2021_S1+S2_90.zip	Merged data measured by MS-711 and 712 (300–1700 nm) during 2021 at 90° divided in two files: Jan to Jun; and Jul to Dec.

2022	35	2022_S1_35.zip	Data measured by MS-711 (300–1100 nm) during 2022 at 35° divided in three files: Jan to Apr; May to Oct; and Sep to Dec.
		2022_S2_35.zip	Data measured by MS-712 (900–1700 nm) during 2022 at 35° divided in three files: Jan to Apr; May to Oct; and Sep to Dec.
		2022_S1+S2_35.zip	Merged data measured by MS-711 and 712 (300–1700 nm) during 2022 at 35° divided in three files: Jan to Apr; May to Oct; and Sep to Dec.

2022	90	2022_S1_90.zip	Data measured by MS-711 (300–1100 nm) during 2022 at 90° divided in three files: Jan to Apr; May to Oct; and Sep to Dec.
		2022_S2_90.zip	Data measured by MS-712 (900–1700 nm) during 2022 at 90° divided in three files: Jan to Apr; May to Oct; and Sep to Dec.
		2022_S1+S2_90.zip	Merged data measured by MS-711 and 712 (300–1700 nm) during 2022 at 90° divided in three files: Jan to Apr; May to Oct; and Sep to Dec.

## 3 Experimental Design, Materials and Methods

The data was acquired using pairs of spectrometers installed outdoors on top of a building in Berlin (52°25′52.5″N 13°31′25.7″E) on a metallic rack with two different tilt angles (See Fig. 1).

The spectrometers were provided by *EKO-instruments* [2]. The first spectrometer is the model MS-711, which measures spectral irradiance within a range of 300–1100 nm. The second spectrometer is the model MS-712 with a measurement range of 900–1700 nm. The spectrometers have a wavelength accuracy of ±0.2 nm. Both spectrometers have an optical (wavelength) resolution (FWHM) better than 7 nm and both spectrometers possess a field of view of 180°. For the acquisition of this data the integration time was set to 5 min intervals. The data was merged automatically by software provided by *EKO-instruments (i.e. WSDac, WSDisp).*

## Ethics Statements

All authors comply with the ethical guidelines contained in Data in Brief's Guide for Authors.

The data was obtained without any involvement of human subjects, animals, or social media platforms.

## CRediT Author Statement

**Guillermo A. Farias-Basulto:** Visualization, Draft preparation, Writing – review & editing; **Maximillian Riedel:** Methodology, Software, Data Curation, Validation; **Mark Khenkin:** Methodology, Writing – review & editing; **Rutger Schlatmann:** Supervision, Resources; **Reiner Klenk:** Writing – review & editing; **Carolin Ulbrich:** Methodology, Project administration, Funding acquisition, Reviewing, Supervision, Resources.

## Declaration of Competing Interest

The authors declare that they have no known competing financial interests or personal relationships that could have appeared to influence the work reported in this paper.

## Data Availability

Solar spectra datasets at optimum and vertical installation angles in central Europe (Berlin) during 2020, 2021 and 2022 (Original data) (HZB Data Service).
